# The Effect of Expressive Writing on the Experiences of Head and Neck Cancer Patients Undergoing Radiotherapy: A Randomized Controlled Trial

**DOI:** 10.1002/cam4.70595

**Published:** 2025-01-09

**Authors:** Jiayuan Li, Zhuoran Gao, Siyu Li, Xia Zhong

**Affiliations:** ^1^ Department of the First Clinical Medical College Jinzhou Medical University Jinzhou China; ^2^ Department of Radiation Oncology The First Hospital of China Medical University Shenyang China; ^3^ Department of Orthopedics Qilu Hospital of Shandong University Jinan Shandong People's Republic of China

**Keywords:** expressive writing, head and neck cancer, mental health, nutritional status, sleep quality

## Abstract

**Background:**

Expressive writing (EW) has emerged as an innovative strategy for improving mood and quality of life. Nevertheless, insufficient research has been conducted on the impact of offering EW to patients with HNC. Therefore, the purpose of this study was to investigate the effects of two forms of EW on anxiety, depression, nutrition, and sleep quality in HNC patients undergoing radiotherapy.

**Methods:**

We conducted a single‐blind, pretest, posttest, three‐group randomized controlled trial. A total of 147 patients with HNC were randomly assigned to a benefit‐finding writing group, neutral writing group, or control group. The intervention group patients performed EW during radiotherapy, with each writing session lasting 20 min, once a week for 4 consecutive weeks. Patient anxiety, depression, nutritional status, and sleep quality were measured at baseline (T0) and at the end of radiotherapy (T1).

**Results:**

After 4 weeks of intervention, patients in the BF and NW groups experienced improvements in anxiety, depression, and sleep (*p* < 0.05) compared with those in the CG group, but the intervention did not significantly affect patients' nutritional status (*p* > 0.05). Compared with those in the CG, anxiety in the BF and NW groups slowed down the trend of increasing anxiety, and repeated measures analysis revealed a significant group × time interaction (*p* = 0.017, *F* = 4.205, *η* ^2^ = 0.059). Compared with those in the CG, the depression levels in the BF and NW groups were lower than those at baseline, and repeated measurement analysis revealed that the interaction effect between group × time was significant (*p* = 0.000, F = 16.262, *η* ^2^ = 0.194). The sleep quality in the CG progressively worsened from T0 to T1 (*p* < 0.01), whereas in the BF, it progressively improved (*p* < 0.01).

**Conclusions:**

This study provides preliminary evidence that two forms of EW are effective in alleviating anxiety and depression and improving sleep in patients with HNC but are not effective in improving their nutritional status.

**Trial Registration:** ChiCTR2400084964

**Abbreviations**
BF: benefit‐finding writing group
CG: control group
HADS: Hospital Anxiety and Depression Scale
HNC: head and neck cancer
IMRT: intensity‐modulated radiotherapy
NW: neutral writing group
PG‐SGA: Subjective Gross Nutrition Assessment Scale
PSQI: Pittsburgh Sleep Quality Index

## 1 Background

Cancer is a major public health problem worldwide, and its incidence and mortality rates are rapidly increasing. Head and neck cancer (HNC), a common malignant tumor, accounts for approximately 10%–30% of systemic malignant tumors and ranks sixth in the world [1]. According to the 2020 Malignant Tumor Statistical Report [1, 2], there were 19.29 million new cases of malignant tumors in the world in 2020, including 750,000 cases of HNC. There are 4.57 million new cases of malignant tumors in China, including 142,000 cases of HNC, which is a common malignant tumor in China. In recent years, with the continuous development of diagnostic and therapeutic techniques, the 5‐year survival rate of patients with HNC has improved significantly, from 54.1% to 66.8% [3]. As a result, people are paying increasing attention to quality of life, especially the development of their physical and mental health.

However, for most patients with HNC, a disease diagnosis may be a traumatic event or a near‐death health condition [4]. It typically causes patients to experience posttraumatic stress disorder, which then triggers psychological consequences (e.g., anxiety, depression) and somatic symptoms (e.g., sleep disturbances and malnutrition) throughout the entire cancer disease course [5]. Comprehensive therapy based on intensity‐modulated radiotherapy (IMRT) has become the primary treatment for most HNC patients, which not only accurately treats cancer and slows the progression of the tumor but also improves the survival rate of patients [6].

However, radiotherapy also produces a series of complications such as pain, oral mucositis, dysphagia, eating difficulty, and psychoneurological symptoms such as cognitive dysfunction, anxiety, and depression, which can lead to malnutrition and restriction in daily activities in patients with HNC [7, 8, 9]. In HNC patients undergoing IMRT, anxiety and depression are important indicators for comparing treatment outcomes, and anxiety and depression are associated with sleep disturbances [9]. Related studies have shown that psychological distress (depression and anxiety) and sleep disorders impact patients' disease rehabilitation and long‐term quality of life [10], thereby affecting occupational, social, and interpersonal functioning in patients with HNC [11]. In addition, malnutrition in patients affects treatment efficacy, which is closely related to recurrent disease and unnecessary medical expenses [12].

Although there are currently methods of drug intervention to reduce anxiety, depression, and sleep quality in patients, drug treatment has side effects [13]. Health professionals have also conducted various nonpharmacological interventions, such as muscle relaxation and physical exercise, to address psychological distress and sleep quality [11, 14]. While these therapies are acceptable and beneficial, it is unclear whether individuals with HNC can sustain long‐term results by adhering to the research protocol only passively. In addition, the radiotherapy of cancer patients is an important phase of the patient's treatment period, during which patients are prone to a variety of complications [15]. Several studies have demonstrated the beneficial effects of early preventative measures on the prevention of problems in patients receiving radiation [16, 17]. Therefore, from the perspective of patient concern and interest, adjusting and managing the psychological distress, nutritional status, and sleep status of HNC radiotherapy patients is a difficult and complex challenge.

Expressive writing (EW), also known as written emotional disclosure, is a psychological intervention model proposed by Pennebaker et al. [18] in the 1990s. It has been proven to be effective in preventing cognitive and emotional changes in cancer patients after diagnosis [19] and can ensure physical and mental health to some extent [20, 21]. The mechanism behind this is that by writing down their thoughts and feelings, patients can actively express feelings and thoughts related to personally significant experiences or positive events, explore the experiential and creative significance of coherent narratives, and ultimately actively benefit from the experience in a self‐care manner rather than passively receiving it [22]. On the basis of the content of the writing, EW can be divided into four categories: free‐choice writing, benefit‐finding writing (i.e., writing about positive thoughts and feelings), neutral writing (i.e., writing about their daily activities or non‐emotional facts about their cancer), and emotional writing (i.e., writing about their emotions about their cancer) [23]. A systematic review verified the enduring influence of EW on patients' stress, anxiety, and depression [24]. According to the quantitative findings of a meta‐analysis review [23], the nonwriting control group had a greater impact on physical and mental health than did the neutral writing group. Furthermore, the effects of benefit‐finding writing have not received much research attention. Although EW is widely accepted among oncology patients, its use in the HNC population is limited [25], and the majority of pertinent research is presently concentrated on patients with breast cancer [26]. HNC in patients during radiotherapy, due to the effect of tracheal intubation, which results in patient pronunciation difficulties and language expression, has certain obstacles, coupled with patients' difficulties eating, reduced appetite, body image changes, and other complications, and patients have a greater need for self‐regulation through expressive writing.

Considering that once EW has been shown to have a significant effect on HNC patients, it is not only a low‐cost alternative to self‐management procedures but also saves time and improves the efficiency of communication between healthcare professionals and HNC patients, reduces the invisible workload of healthcare professionals and improves medical efficiency. Therefore, this study aimed to investigate the effects of two interventions—benefit‐finding writing and neutral writing—on the mental and physical health of patients with HNC receiving radiation treatment. We hypothesized that both benefit‐finding writing and neutral writing would reduce patients' anxiety and depression, improve sleep quality and improve nutritional status. In addition, benefit‐finding writing had a more significant effect than neutral writing did.

## 2 Methods

### 2.1 Study Design

This parallel, three‐arm randomized controlled trial with a prospective, single‐blind (outcome assessor‐blinded), pretrial, and posttrial design was conducted at the National Cancer Regional Medical Center, a tertiary hospital in northeastern China. This study was approved by the Institutional Review Ethics Committee of the First Hospital of China Medical University (approval number: [2024] 495). Written informed consent was obtained. Data privacy was guaranteed. Participants could leave the study at any time, according to the Declaration of Helsinki. The study was reported in accordance with the Consolidated Standards for Reporting Trials (CONSORT) 2010 guidelines.

### 2.2 Participants

Patients with newly diagnosed head and neck neoplasms who were prepared for their first radiotherapy session were enrolled from June to November 2023. The inclusion criteria for this study were as follows: (1) had a new diagnosis of HNC by pathology or histology, (2) were aged ≥ 18 years, (3) understood their condition and were receiving radiotherapy, (4) were able to write diary contents, and (5) provided informed consent and voluntary consent. The exclusion criteria were as follows: (1) had a history or diagnosis of cognitive impairment, (2) had comorbidities of other neoplasms or other major diseases, (3) had received psychological counseling and psychotherapy and (4) had recently experienced other negative events.

### 2.3 Procedure

#### 2.3.1 Sample Size Calculation

In a previous comparative study, the sample size was calculated on the basis of the changes in depressive symptoms after 3 months of intervention. According to this study, the effect size of depressive symptoms in the benefit‐finding writing group was 0.76 [27]. Using G*power v.3.1.9.2 software, we calculated that 39 patients in each group needed 80% power and a bilateral 0.05 *α* level test to detect differences. Considering a 20% attrition rate, we aimed to recruit a total of 147 participants, with 49 participants in each group.

#### 2.3.2 Randomization, Concealment, and Blindness

After the baseline assessment, patients were randomly assigned to the benefit‐finding writing group (BF), neutral writing group (NW), or control group (CG). Considering that participants were recruited continuously with the progress of the experiment rather than at a certain point in time, a permuted random block allocation method (block size: 6) was used to ensure equal sample sizes between groups over time. The computer‐generated random assignment sequence via Excel was controlled by an independent researcher (G) who was not involved in participant recruitment, outcome assessment, or the expressive writing intervention. Assignment concealment was ensured by using sealed opaque envelopes distributed by one researcher (Z). Whenever 6 patients were successfully recruited, the randomization procedure (block size = 6) was assigned at a ratio of 1:1:1 (i.e., 2 patients were allocated to each of the BF, NW, or CG). Primary and secondary outcomes were assessed at baseline and at the completion of the 4‐week writing period. All the data collectors (X and L) were unaware of the group assignment. Owing to the nature of the intervention, it was impossible to blind participants and the nursing researchers who conducted the intervention. However, the fact that the data collectors and data analysts were unaware of the grouping of patients ensured that the results were more accurate.

#### 2.3.3 Interventions

Each participant in the BF and NW groups was instructed by the researcher (Z) to write once a week for 20 min, continuously for 4 weeks. (a) Write down any positive thoughts and feelings about the experience of having head and neck neoplasm (BF). The complete explanation is as follows: What we want you to do is write down any positive thoughts and feelings about your experience of having HNC. We know that patients with HNC will experience various emotions, but we hope you will focus on the positive emotions, thoughts, and life changes you have experienced. For example, some patients feel that they have learned important life lessons from their experience with cancer. You can also connect your positive thoughts and feelings about your cancer experience with other parts of your life—your childhood, the person you love, who you want to be (please use names only), or what you want to do after discharge/recovery. Ideally, we want you to write for 15–20 min without interruption. If you have nothing to say, repeat what you have already written until the end of the 20 min. Do not worry about grammar.

Write down (b) their daily life, treatment process, or facts unrelated to emotions (NW), such as dietary behavior, exercise behavior, physical activity, and sleep habits. To guarantee that they had enough time to write without interruption, participants were advised ahead of time about the expected writing circumstances, which included turning off all electronic devices and being alone in the room in a comfortable spot. The researcher (Z) will meet with each patient every week in the radiation therapy room to supervise the study participants in completing their writing assignments and to retrieve the previous week's writing for checking. If the diary was not written correctly, all patients received phone follow‐up visits. When the 4‐week writing was completed, patients returned face‐to‐face to their last completed writing in the radiotherapy treatment room.

The control group participants received routine care (including health education, observation, and psychological care) from professionally trained nurses. A qualified oncologist provided them with advice regarding nutrition, consistent exercise, rehabilitation exercises, regular follow‐up appointments, and attitudes regarding the use of alcohol and cigarettes, among other pertinent topics.

### 2.4 Outcome Measurements

The primary outcome was psychological distress, including depression and anxiety. The secondary outcomes included nutritional status and sleep quality. Two data collectors (J and S, unaware of patient allocation) collected data through face–to–face interviews and observations of patients in wards or radiotherapy treatment rooms. HNC survivors completed the HADS, PG‐SGA, and PSQI at baseline (T0) and at the end of radiotherapy (T1).

The general information questionnaire included general demographic and sociological information (gender, age, education level, marital status, etc.) and disease‐related information (smoking history, drinking history, type of cancer, treatment modality, disease duration, weight, etc.) extracted by the researcher from the patient's medical records.

Patients' psychological distress was measured via the 14‐item Hospital Anxiety Depression Scale (HADS) [28]. This scale mainly assesses emotional status in the past month. The higher the score is, the more severe the anxiety or depression. It contains two subscales, the Anxiety Symptoms Scale (HADS‐A; 7 items) and the Depressive Symptoms Scale (HADS‐D; 7 items), with each subscale scoring 0–3 points and each subscale scoring a total of 0–21 points. The Cronbach's α coefficient of the total scale is 0.879, and the Cronbach's *α* coefficients of the anxiety and depression subscales are 0.80 each.

Nutritional status was measured via the PG‐SGA [29]. The PG‐SGA consists of two parts: the first part is filled in by the patient's family members, whereas the second part is filled in by the nurses after they assess the patient's actual condition. The final score is obtained by summing the scores of the two parts, and the higher the score is, the worse the nutritional status. The content of the PG‐SGA reflects recent changes in the patient's weight, the nutritional requirements of the disease, and the storage of muscle and fat in the body.

Patients' sleep quality in the past month was assessed by the Pittsburgh Sleep Quality Index (PSQI). The scale was developed by Dr. Buysse et al. [30] at the University of Pittsburgh to assess patients' sleep quality and disturbance status. Liu Xianchen et al. [31] conducted the PSQI scale localization and reliability and validity tests, and the results showed that the Cronbach's α coefficient of the PSQI scale was 0.84. The scale consists of 23 entries and is divided into 7 parts, namely, subjective sleep quality, time to sleep, sleep efficiency, sleep duration, sleep disorders, hypnotic medications, and daytime dysfunction. The scale uses a 4‐point scoring method, and the sum of the scores of each part is the total PSQI score. The higher the score is, the worse the patient's sleep condition.

### 2.5 Statistical Analysis

This study used Excel to input the experimental data and SPSS 26.0 for the statistical analysis (IBM Corp., Armonk, NY, USA). Normally distributed quantitative data are expressed as the mean ± standard deviation, and counting data are presented as frequencies and percentages. One‐way ANOVA or chi‐square tests were used to compare sociodemographic data and key outcome variables across groups. A mixed model ANOVA with post hoc Bonferroni adjustment was used to determine the effects of time, group, and group × time interactions. Effect sizes for the mixed‐measures ANOVA were calculated as skewed variance (*η* ^2^), categorized as small 0.02 ~ 0.13, medium 0.13 ~ 0.26, and large > 0.26. *p* < 0.05 was considered to indicate statistical significance.

## 3 Results

### 3.1 Participant Flow

**FIGURE 1:**
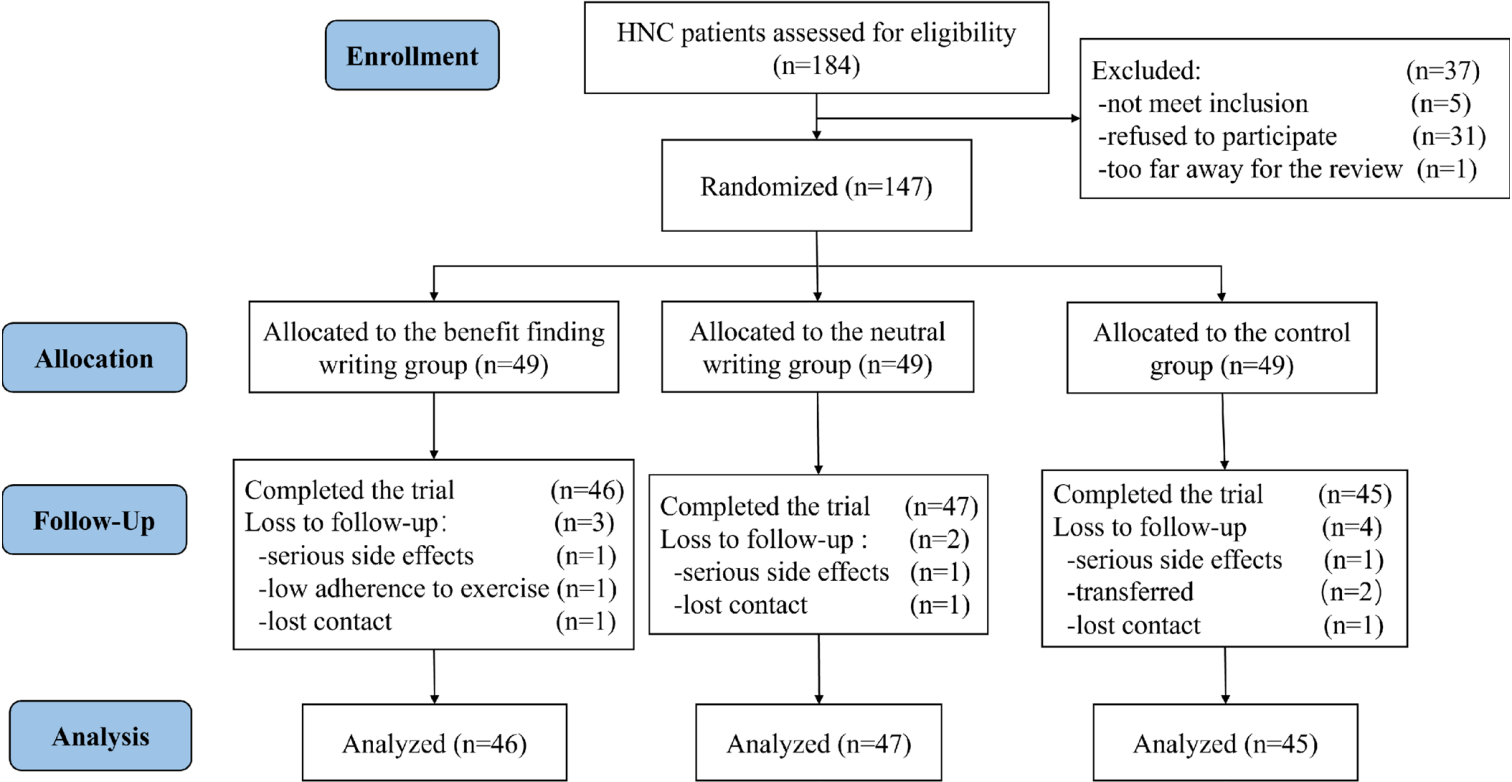
Flowchart of patient recruitment, randomization, and attrition.

A total of 184 HNC patients who received radiotherapy underwent qualification evaluation, 147 of whom agreed to participate. The participants were randomly assigned to the BF (*n* = 49), NW (*n* = 49), or CG (*n* = 49) group. After 4 weeks, 3 participants (93.88%, 46/49) dropped out of BF, 2 participants (95.92%, 47/49) dropped out of NW, and 4 participants (91.84%, 45/49) dropped out of CG. Ultimately, 138 participants were included in the analysis (Figure 1). There was no statistically significant difference in the proportion of patients who withdrew from the three groups. No adverse events occurred during the entire intervention process.

### 3.2 Baseline Characteristics

A total of 138 patients with an average age of 55.10 ± 9.23 years were included. Among them, 110 were male, accounting for 79.7%. There was no statistically significant difference among the three groups in terms of demographic information, disease‐related information, anxiety and depression, nutritional status, or sleep quality (*p* > 0.05) (Table 1).

**TABLE 1:** Participants' characteristics and outcome variables at baseline (*n* = 138).

Variable	Total (*n* = 138)	BF (*n* = 46)	NW (*n* = 47)	CG (*n* = 45)	*𝛘* ^2^/*F*	*p*
Age (mean ± SD)	55.10 ± 9.23	55.67 ± 7.55	54.74 ± 9.04	54.89 ± 11.01	0.134^a^	0.875
Gender					0.956^b^	0.617
Male	110 (79.7%)	36 (78.3%)	36 (76.6%)	38 (84.4%)		
Female	28 (20.3%)	10 (21.7%)	11 (23.4%)	7 (15.6%)		
Family residence					0.047^b^	0.977
City	72 (52.2%)	24 (52.2%)	24 (51.1%)	24 (53.3%)		
Countryside	66 (47.8%)	22 (47.8%)	23 (48.9%)	21 (46.7%)		
Type of cancer					5.798^b^	0.97^c^
Cancer of the oral cavity	16 (11.6%)	6 (13.0%)	5 (10.6%)	5 (11.1%)		
Cancer of the nasopharynx	66 (47.8%)	21 (45.7%)	24 (51.1%)	21 (46.7%)		
Cancer of the oropharynx	20 (14.5)	7 (15.2%)	7 (14.9%)	6 (13.3%)		
Cancer of the hypopharynx	19 (13.8%)	6 (13.0%)	6 (12.8%)	7 (15.6%)		
Cancer of the larynx	7 (5.1%)	2 (4.3%)	1 (2.1%)	4 (8.9%)		
Ethmoid sinus and maxillary sinus neoplasms	6 (4.3%)	2 (4.3%)	2 (4.3%)	2 (4.4%)		
Occult primary neoplasms	2 (1.4%)	1 (2.2%)	1 (2.1%)	0 (0%)		
Large salivary gland cancer	2 (1.4%)	1 (2.2%)	1 (2.1%)	0 (0%)		
Education level (years)					8.254^b^	0.220^c^
≤ 6	14 (10.1%)	6 (13.0%)	4 (8.5%)	4 (8.9%)		
7–9	50 (36.2%)	19 (41.3%)	17 (36.2%)	14 (31.1%)		
10–12	41 (29.7%)	13 (28.3%)	18 (38.3%)	10 (22.2%)		
13–15	33 (23.9%)	8 (17.4%)	8 (17.0%)	17 (37.8%)		
Disease stage					0.537^b^	0.970^c^
II and below	3 (2.1)	1 (2.2%)	1 (2.1%)	1 (2.2%)		
III	32 (23.2%)	9 (19.6%)	12 (25.5%)	11 (24.4%)		
IV	103 (74.7%)	36 (78.3%)	34 (72.3%)	33 (73.4%)		
Surgery					0.098^b^	0.952
No	71 (51.4%)	23 (50.0%)	25 (53.2%)	23 (51.1%)		
Yes	67 (48.6%)	23 (50.0%)	22 (46.8%)	22 (48.9%)		
Chemotherapy	138				7.741^b^	0.255^c^
No	18 (13.0%)	4 (8.7%)	6 (12.8%)	8 (17.8%)		
Induction chemotherapy (IC)	14 (10.2%)	3 (6.5%)	5 (10.6%)	6 (13.3%)		
Concomitant chemotherapy (CC)	57 (41.3%)	21 (45.7%)	15 (31.9%)	21 (46.7%)		
IC + CC	49 (35.5%)	18 (39.1%)	21 (44.7%)	10 (22.2%)		
The number of comorbidities					7.409^b^	0.542^c^
0	68 (49.3%)	24 (52.2%)	20 (42.6%)	24 (53.3%)		
1	41 (29.7%)	13 (28.3%)	16 (34.0%)	12 (26.7%)		
2	21 (15.2%)	8 (17.4%)	8 (17.0%)	5 (11.1%)		
3	6 (4.3%)	1 (2.2%)	3 (6.4%)	2 (4.4%)		
4	2 (1.4%)	0 (0%)	0 (0%)	2 (4.4%)		
Smoking history					5.047^b^	0.080
No	58 (42.0%)	8 (39.1%)	21 (44.7%)	19 (42.2%)		
Yes	80 (58.0%)	28 (60.9%)	26 (55.3%)	26 (57.8%)		
Drinking history					3.744^b^	0.154
No	76 (55.1%)	18 (39.1%)	27 (57.4%)	25 (55.6%)		
Yes	62 (44.9%)	28 (60.9%)	20 (42.6%)	20 (44.4%)		
HADS‐A		4.15 ± 2.49	4.53 ± 2.48	4.96 ± 3.42	0.920^a^	0.401
HADS‐D		7.37 ± 3.32	7.79 ± 3.32	7.22 ± 2.97	0.387^a^	0.680
PG‐SGA		3.33 ± 2.56	3.13 ± 2.37	3.20 ± 1.80	0.091^a^	0.913
BMI		23.88 ± 3.16	24.19 ± 3.51	23.40 ± 3.69	0.605^a^	0.548
PSQI		5.00 ± 2.29	4.47 ± 2.00	5.27 ± 2.93	1.294^a^	0.277

Abbreviations: BF, benefit‐finding writing group; CG, control group; NW, neutral writing group; SD, standard deviation.

^a^ ANOVA.

^b^ Chi‐squared test.

^c^ Because the expected count of cells was less than 5, the *p*‐value was determined by Fisher's exact test rather than the Pearson chi‐squared test.

### 3.3 Primary and Secondary Outcomes

**FIGURE 2:**
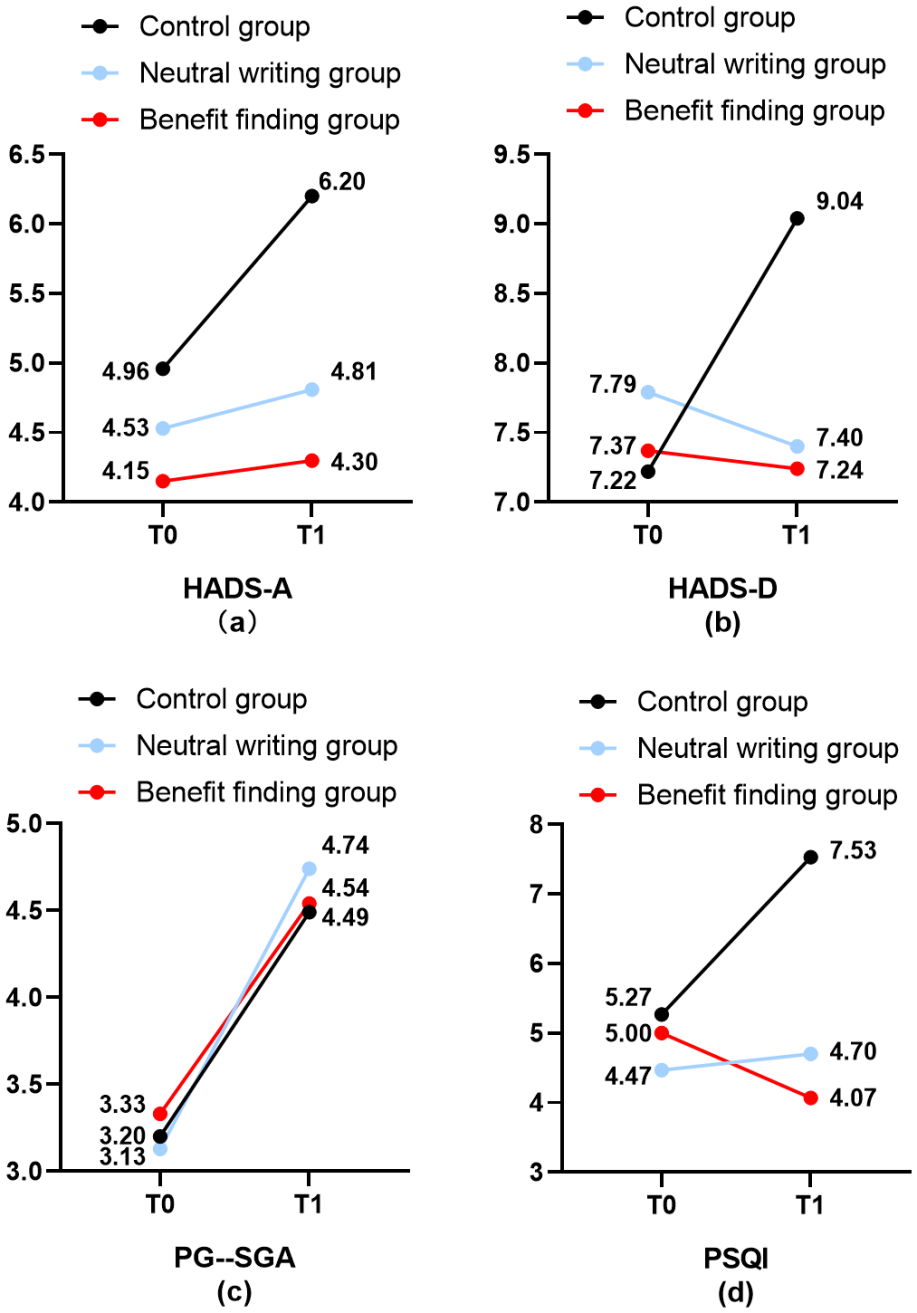
Changes in levels of anxiety, depression, malnutrition, and sleep at overall time points according to the group.

Table 2 and Figure 2 demonstrate the changes in anxiety, depression, nutrition, and sleep status from T0 to T1 in the three groups of patients.

**TABLE 2:** Repeated measures for primary and secondary outcomes in the groups at baseline and postintervention.

Outcome variables	BF (mean ± SD)	NW (mean ± SD)	CG (mean ± SD)	Comparison of mean difference in change at 4th week									Mixed design repeated measures ANOVA test		
				BF versus NW			BF versus CG			NW versus CG					
				*d*	*p*	95% CI	*d*	*p*	95% CI	*d*	*p*	95% CI	*F*	*η* ^2^	*p*
HADS‐A															
T0	4.15 ± 2.49	4.53 ± 2.48	4.96 ± 3.42	−0.380	1.000	−1.800 to 1.041	−0.803	0.532	−2.239 to 0.633	−0.424	1.000	−1.852 to 1.005	11.106	0.076	0.001^a^ ^,^ **
T1	4.30 ± 2.10	4.81 ± 2.48	6.20 ± 3.03	−0.504	1.000	−1.791 to 0.783	−1.896	0.002**	−3.196 to −0.595	−1.391	0.030*	−2.685 to −0.098	3.413	0.048	0.036^b^ ^,^ *
*d*	−0.152	−0.277	−1.244										4.205	0.059	0.017^c^ ^,^ *
*p*	0.600	0.336	0.000**												
HADS‐D															
T0	7.37 ± 3.32	7.79 ± 3.32	7.22 ± 2.97	−0.418	1.000	−2.030 to 1.195	0.147	1.000	−1.483 to 1.777	0.565	1.000	−1.056 to 2.186	6.343	0.045	0.012^a^ ^,^ *
T1	7.24 ± 3.14	7.40 ± 3.37	9.04 ± 3.11	−0.165	1.000	−1.781 to 1.450	−1.805	0.025*	−3.438 to −0.172	−1.640	0.047*	−3.265 to‐0.016	0.865	0.013	0.423^b^
*d*	0.130	0.383	−1.822										16.262	0.194	0.000^c^ ^,^ **
*p*	0.662	0.196	0.000**												
PG‐SGA															
T0	3.33 ± 2.56	3.13 ± 2.37	3.20 ± 1.80	0.198	1.000	−0.944 to 1.340	0.126	1.000	−1.028 to 1.281	−0.072	1.000	−1.221 to 1.076	81.786	0.377	0.000^a^ ^,^ **
T1	4.54 ± 2.42	4.74 ± 2.18	4.49 ± 1.60	−0.201	1.000	−1.257 to 0.855	0.055	1.000	−1.013 to 1.122	0.256	1.000	−0.806 to 1.318	0.032	0.796	0.969^b^
*d*	−1.217*	−1.617*	−1.289*										0.664	0.010	0.517^c^
*p*	0.000**	0.000**	0.000**												
BMI															
T0	23.88 ± 3.16	24.19 ± 3.51	23.40 ± 3.69	−0.308	1.000	−2.049 to 1.433	0.481	1.000	−1.279 to 2.241	0.789	0.830	−0.962 to 2.540	37.876	0.219	0.000^a^ ^,^ **
T1	22.06 ± 2.78	22.35 ± 3.19	22.22 ± 3.72	−0.284	1.000	−1.917 to 1.350	−0.233	1.000	−1.812 to 1.491	0.123	1.000	−1.520 to 1.766	0.278	0.004	0.758^b^
*d*	1.820	1.844	1.178										0.684	0.010	0.506^c^
*p*	0.000**	0.000**	0.011*												
PSQI															
T0	5.00 ± 2.29	4.47 ± 2.00	5.27 ± 2.93	0.532	0.879	−0.689 to 1.753	−0.267	1.000	−1.501 to 0.968	−0.799	0.352	−2.027 to 0.430	6.784	0.048	0.010^a^ ^,^ *
T1	4.07 ± 1.93	4.70 ± 2.00	7.53 ± 2.34	−0.637	0.436	−1.691 to 0.418	−3.468	0.000**	−4.534 to −2.402	−2.831	0.000**	−3.892 to −1.771	13.659	0.168	0.000^b^ ^,^ **
*d*	0.935	−0.234	−2.267										21.518	0.242	0.000^c^ ^,^ **
*p*	0.008**	0.497	0.000**												

Abbreviations: BF, benefit‐finding writing group; CG, control group; *d*: mean difference = T0–T1; NW, neutral writing group; SD, standard deviation.

^a^ Time effect.

^b^ Group effect.

^c^ Group×time effect.

* *p* < 0.05.

** *p <* 0.01.

Mixed‐design repeated‐measures ANOVAs revealed significant time effects for anxiety and depression in the BF, NW, and CG groups (*p* = 0.001, *F* = 11.106, *η* ^2^ = 0.076; *p* = 0.012, *F* = 6.343, *η* ^2^ = 0.045). Repeated‐measures group × time interactions revealed significant differences in levels of anxiety and depression among the groups (*p* = 0.017, *F* = 4.205, *η* ^2^ = 0.059, and *p* = 0.000, *F* = 16.262, *η* ^2^ = 0.194). A pairwise comparison was conducted between groups, and the differences between the BF and CG (anxiety: *p* = 0.002; depression: *p* = 0.025) and between the NW and CG (anxiety: *p* = 0.030; depression: *p* = 0.047) were statistically significant. There was no statistically significant difference in BF compared to NW (anxiety: *p* = 1.000; depression: *p* = 1.000), indicating that there was no difference in anxiety and depression changes between the intervention groups.

For nutrition and BMI, the interaction between time and group was not significant (*p* = 0.969, *F* = 0.032, *η* ^2^ = 0.796; *p* = 0.758, *F* = 0.278, *η* ^2^ = 0.004), the interaction between time× group was not significant (*p* = 0.517, *F* = 0.664, *η* ^2^ = 0.010; *p* = 0.506, *F* = 0.684, *η* ^2^ = 0.010), but the main time effect was significant (*p* = 0.000, *F* = 81.786, *η* ^2^ = 0.377; *p* = 0.000, *F* = 37.876, *η* ^2^ = 0.219), indicating that the nutritional status of patients gradually decreased with the beginning of radiotherapy. Neither BF nor NW expression affected the nutritional status of the patients. For sleep, the main effect of time, the main effect of the group, and the interaction between time × group were significant (*p* = 0.010, *F* = 6.784, *η* ^2^ = 0.048; *p* = 0.000, *F* = 13.659, *η* ^2^ = 0.168; *p* = 0.000, *F* = 21.518, *η* ^2^ = 0.242, respectively). However, there was no difference in the impact of BF or NW on the sleep quality of patients (*p* = 0.436).

## 4 Discussion

This study investigated the symptoms and needs of patients with HNC during radiotherapy, considering that adverse effects such as mood changes and complications during radiotherapy alter patients' sleep and affect their nutritional status. In addition, it hinders patient communication because of the effects of tracheal intubation [32]. Therefore, HNC patients may benefit from using writing to express their emotions so that they can directly participate in the disease treatment process and stimulate their subjective initiative. Our findings suggest that EW can improve anxiety, depression, and sleep quality in HNC patients to some extent, regardless of the type of EW intervention. This finding is consistent with the results of a study conducted by Jing Wang [33]. Therefore, by writing about their positive or neutral thoughts, patients can take the initiative to identify their own unhealthy or negative thinking and behavioral patterns, improve their self‐awareness, and then take their subjective initiative to improve their quality of life.

This study compared the anxiety and depression levels of three groups of patients and revealed that, compared with the CG, both the BF and NW groups had significantly lower levels of anxiety and depression. Kerry [34] came to the same conclusion that EW can alleviate depression in breast cancer patients. This finding is also similar to the findings of Eldesouky [35] that EW in the form of benefit discovery encourages individuals to establish positive, supportive, and comprehensive self‐dialog, which can improve patients' anxiety‐depressive mood to some extent. Consequently, our research provides additional support for the supplemental therapeutic benefit of EW in patients undergoing radiation and experiencing mental health issues such as anxiety and depression [36]. However, the findings of a meta‐analysis of EW intervention studies in early health and clinical samples [24, 37] concluded that writing their treatment and experience in terms of emotions had little positive impact on their physical or mental well‐being. More trials are needed to validate the effectiveness of EW on patients' mental health. There was no difference between the BF and NW groups in terms of anxiety and depression; that is, regardless of the writing method, the anxiety and depression of patients decreased. Therefore, language use patterns (such as the use of emotional words) during EW do not mediate the impact of emotional expression on health outcomes [26]. In terms of numerical values, the anxiety and depression scores of patients in the BF were slightly lower than those in the NW. Therefore, clinical medical staff should choose the most suitable writing method for each patient on the basis of their preferences.

Our study revealed that with the start of radiation therapy, anxiety and depression gradually increase in patients with HNC. This may be related to the introverted personalities of Chinese people, who are not good at expressing themselves and who usually suppress their negative emotions deep in their hearts, thereby increasing their psychological burden. These findings suggest that medical staff should focus on patients who are receiving radiotherapy. In this study, patients expressed negative emotions through EW, recorded their experiences, explored their inner selves, and expressed their suppressed emotions. This promoted the expression of their emotions and the organization of their thinking and alleviated anxiety and depression during radiotherapy. On the other hand, it also utilizes the power of writing to guide patients in examining negative thinking patterns, challenging impossible beliefs, and cultivating positive thinking and behavioral habits. The positive thinking of patients can play an important role in health management and rehabilitation processes [38]. Previous studies have shown that patients with HNC who have a positive attitude have greater compliance with treatment plans [39]. Therefore, it is necessary to promote positive thinking in patients with HNC. It is recommended that clinical medical staff pay attention not only to the treatment effectiveness of patients with HNC but also to their psychological state during radiotherapy, identify negative emotional danger signals early, carry out relevant psychological counseling work, guide patients to vent their negative emotions and lead patients to engage in positive thinking.

However, after the intervention in this study, there was no statistically significant difference in the nutritional status of the three groups of patients, indicating that neither BF nor NW improved the nutritional status of patients during radiotherapy. Furthermore, previous studies on EW have not explored whether it has an effect on nutritional status. Therefore, future studies should also verify the long‐term effects of expressive writing on the nutritional status of patients via the use of more objective nutritional indicators. The nutritional status of all three groups of patients deteriorates with the progression of radiotherapy, which may be related to complications such as oral mucosal inflammation and eating difficulties that occur during radiotherapy, leading to a low appetite. Therefore, medical staff should focus on the nutritional status of patients with HNC during radiotherapy. Screening and evaluation are the first steps in nutritional management. At the same time, timely nutritional support monitoring and care should be provided for patients with malnutrition [40].

This study revealed that both BF and NW improved sleep quality in patients with HNC by guiding patients to think about their positive thoughts after their positive thoughts or objective experiences were written on paper, which is consistent with the results of Liu's study on the effect of EW on positive psychological interventions on the quality of sleep in patients with hepatocellular carcinoma [41]. Interestingly, in the CG, the sleep score gradually increased with increasing radiotherapy, indicating that the patient's sleep quality gradually deteriorated. In the NW group, the sleep score slightly increased. Only in the BF did the sleep score showed a downward trend, indicating that the patient's sleep quality significantly improved. However, there was no significant difference between the two intervention methods in terms of improvement in patients' sleep quality, which was consistent with the research results on EW in breast cancer patients [42]. The possible reasons for this are as follows: BF patients are found to actively express their inner emotions and thoughts, confide in their inner troubles and anxieties, and fully release their emotions. To a certain extent, this helps reduce stress, relieves tension, promotes physical and mental relaxation, and is more conducive to sleep. However, patients in the NW only record their experiences objectively and do not receive sufficient emotional release, which affects their sleep. However, both writing styles helped patients organize their disorganized thoughts and gradually determined the nature of their problems. By visualizing the problem in words, patients can gain a clearer understanding of what is troubling them and start looking for solutions.

Related studies have shown that high‐quality sleep can help improve cardiac function, relieve fatigue, relax mood, reduce the body's oxygen consumption and the number of complications, and help restore physical strength, which will ultimately be conducive to worsening the disease and recovery [43]. At the same time, the patient's sleep quality improves, the body and mind are relaxed, anxiety is relieved, and the virtuous cycle improves sleep quality. Therefore, clinical healthcare professionals should pay more attention to the sleep quality of patients with HNC during radiotherapy and provide sleep counseling and effective interventions to alleviate the difficulty of falling asleep.

## 5 Study Limitations

There are several limitations to this study. First, all participants were recruited from the same hospital, making it difficult to draw general conclusions that apply to all patients with HNC undergoing radiotherapy. In the future, we encourage studies to confirm the effects of BF and NW in a wider and more representative group of patients with HNC. Second, this study was only conducted for 1 month and failed to determine the long‐term effects of EW on the physical and mental status of the study participants. Further in‐depth studies are needed in the future to explore the long‐term effects of expressive writing interventions. Future research can also consider increasing the number of EWs to investigate the long‐term effects of EW on positive and negative emotions, as well as the improvement effect on interpersonal relationships and the resocialization of patients. This study did not compare the effects of different treatment regimens, radiation doses, analgesic medications, and symptom burdens on patients, and future in‐depth investigations are needed. For the description of patient comorbidities, future studies could use the more authoritative Charlson Comorbidity Index.

## 6 Conclusions

This study suggested that both BF and NW can alleviate anxiety and depression to some extent and improve sleep quality in patients undergoing radiotherapy for HNC but do not affect patients' nutritional status. Although there was no statistically significant difference between the two writing interventions in terms of the nutritional status of patients, the two types of EW have high application value in improving psychology and sleep and are effective and appropriate nonpharmacological interventions to improve the psychology and sleep of radiotherapy patients. It is convenient for patients to carry out and can be effective in preparing patients for early recovery and return to society, which is worthy of being recommended and implemented by oncology nurses in the rehabilitation process of patients with HNC.

## Author Contributions

**Jiayuan Li:** conceptualization; software; writing – original draft. **Zhuoran Gao:** formal analysis; writing – original draft; conceptualization; methodology. **Siyu Li:** investigation; writing – review and editing. **Xia Zhong:** writing – review and editing; formal analysis.

## Ethics Statement

This study was performed in accordance with the principles of the Declaration of Helsinki. This study was approved by the Institutional Review Ethics Committee of the First Hospital of China Medical University (approval number: [2024] 495).

## Consent

All patients signed an informed consent form, all data were assured to remain private, and participants could leave the study at any time.

## Conflicts of Interest

The authors declare no conflicts of interest.

## Data Availability

The datasets used and/or analyzed during the current study are available from the corresponding author upon reasonable request.
